# Comparative study on *Angelica sinensis* after different processing with yellow rice wine in color, aromas, chemical components, and antioxidant activities

**DOI:** 10.1016/j.fochx.2023.100822

**Published:** 2023-08-06

**Authors:** Zhi-Tong Zhang, Yue Jiang, Yali Qi, Huanhuan Guan, Lei Bai, Pan Chen, Wufeng Gao, Guo-Dong Zhuang, Tulin Lu, Guojun Yan

**Affiliations:** a School of Pharmacy, Nanjing University of Chinese Medicine, Jiangsu Engineering Research Center for Development and Application of External Drugs in Traditional Chinese Medicine, Jiangsu Province Engineering Research Center of Classical Prescription, Nanjing 210023, China; b Key Laboratory of Digital Quality Evaluation of Chinese Materia Medica of State Administration of TCM and Engineering & Technology Research Center for Chinese Materia Medica Quality of Guangdong Province, Guangdong Pharmaceutical University, Guangzhou 510006, China

**Keywords:** *Angelica sinensis*, Yellow rice wine processing, Antioxidants, Flash GC e-nose, UPLC-Q-Orbitrap HRMS/MS

## Abstract

This study aimed to explore the differences in raw *Angelica Sinensis* (RAS), wine washing AS (WAS), and wine stir-frying AS (WSAS). The results showed there were differences among the three AS in color and aroma, and 34 aroma compounds were identified. The content determination results revealed the ferulic acid and Z-ligustilide levels of RAS decreased after processing, and those in WAS were higher than in WSAS. Furthermore, 85 representative common components and 37 unique components were tentatively identified in three AS. Finally, the free radical scavenging assay results indicated the antioxidant capacity of RAS was reduced after processing, and the antioxidant capacity of WAS was better than WSAS. Collectively, the RAS undergoes significant changes in color, aromas, components, and antioxidant ability after processing, and the different processing methods also result in significant differences between WAS and WSAS.

## Highlights

• Different processing of RAS induced color and aroma differences between WAS and WSAS.
• The ferulic acid and Z-ligustilide levels in WAS were higher than those in WSAS.
• Different processing of RAS caused component profile differences between WAS and WSAS.
• The antioxidant capacity of WAS was better than WSAS *in vitro.*

## Graphical abstract

**Figure:**
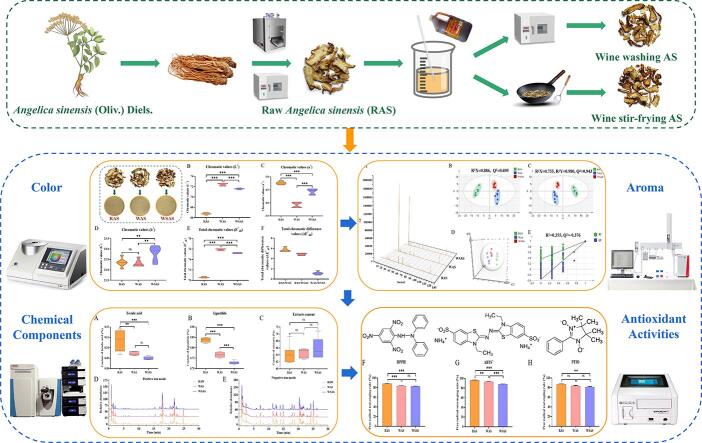

## 1 Introduction

*Angelica sinensis* (AS), also known as Danggui, is widely consumed as a functional food or nutraceutical rich in natural volatile oils, organic acids, and polysaccharides (Long et al., 2022). Recently, many studies have revealed that AS exerts a large variety of biological activities, including anti-arrhythmia, anti-atherosclerosis, anti-oxidation, immunoregulation, and anti-inflammatory, due to its rich active ingredients, such as ferulic acid and Z-ligustilide (Aobulikasimu et al., 2023, Han et al., 2021, Lang et al., 2018). AS has not only traditionally been used as a typical medicine-food herb in the East Asian region for promoting blood circulation, regulating menstruation, reducing pain, and improving constipation, but also used as a dietary supplement for women's care in Europe and North America (Circosta et al., 2006, Dietz et al., 2016, Filipiak-Szok et al., 2014). Notably, traditional Chinese physicians always use different processing methods of AS for different ailments, such as processed with soil, processed with vinegar, and processed with yellow rice wine (Hua et al., 2014). Of these, processing with yellow rice wine is the most common method of AS processing.

Yellow rice wine is a characteristic Chinese wine and one of the three oldest in the world, which has a long history of application in the processing of traditional Chinese medicines (TCM) (Qian et al., 2015). Its unique processing methods can alter the nature and taste of TCM to a certain extent, resulting in major or minor changes in the clinical efficacy of the same TCM before and after processing (Feng et al., 2023, Hu et al., 2022, Wu et al., 2018). The TCM processed with yellow rice wine can rise the medicinal tendency and enhance efficacy, such as activating blood circulation, invigorating Qi, dispelling wind, and dispersing cold (Pei et al., 2022). Modern research has shown that yellow rice wine contains many functional ingredients and has a high nutritional value (Yang et al., 2022a). For AS, before processing with yellow rice wine, it is beneficial to tonify and invigorate the blood, improve constipation, after processing, the smell is changed and the blood circulation and pain relieving effect are enhanced (Yin et al., 2021). Yellow rice wine has been used to process TCM in China historically in many ways, among which the most common and controversial are wine washing and wine stir-frying. Notably, according to historical records, the processing method of *Angelica Sinensis* in many classical prescriptions is wine washing, such as Qingshang Juantong Decoction, Xuanyu Tongjing Decoction, Qinggan Zhilin Decoction, Danggui Buxue Decoction, and Taohong Siwu Decoction, *etc* (Weng et al., 2021, Yang et al., 2021). Moreover, in a recent study, it was found that the therapeutic effect of the wine washing AS (WAS) in Taohong Siwu Decoction was better than the wine stir-frying AS (WSAS), suggesting that there may be a difference between WAS and WSAS (Wang et al., 2022a). However, there is no record regarding WAS in previous editions of the *Chinese Pharmacopoeia* (2020 edition)*.* Comparative studies on the differences among raw AS (RAS), WAS, and WSAS are still lacking.

The spectrophotometer can objectively detect subtle color differences and is widely used in the study of color distribution of herbs and foods (Esatbeyoglu et al., 2023). The Heracles Neo flash GC E-nose is a powerful tool combining e-nose with GC, which can quickly obtain the odor chromatographic information and enable further determine the differential aroma compounds. So it provides excellent differentiation and identification of foods or herbs with differential aromas (Li et al., 2022a). Ultra-performance liquid chromatography quadrupole/electrostatic field orbitrap coupled with high-resolution mass spectrometry (UPLC-Q-Orbitrap HRMS/MS) is an emerging mass spectrometry-based platform, which is fully capable of rapid and accurate characterization of active ingredients in traditional Chinese medicine or foods (Zhang et al., 2021). Thus, combined spectrophotometer, GC E-nose, and UPLC-Q-Orbitrap HRMS/MS analysis provides a new strategy for the study of differences in RAS, WAS, and WSAS.

In this study, we first prepared RAS and then processed the RAS with yellow rice wine in two different ways to obtain the WAS and WSAS. For the three AS, color differences were characterized by spectrophotometry, and aroma profiles were characterized using Heracles Neo flash GC E-nose and screened for differential odors. Then the content of representative components in *Angelica sinensis* was determined, and the chemical profiles of the three AS were further characterized using UPLC-Q-Orbitrap HRMS/MS to screen for their differential chemical composition. Finally, multiple free radical-scavenging assays were measured to compare the biological activity of RAS, WAS, and WSAS. From the perspectives of color, aroma, chemical components, and antioxidant activities, we compared the quality differences among RAS, WAS, and WSAS, which provides a reference for further comparative pharmacological studies and clinical application of *Angelica sinensis* with different processing methods.

## 2 Material and methods

### 2.1 Reagents

Yellow rice wine (batch number 20180905) was purchased from Zhejiang Tapai Shaoxing Wine Co., Ltd. (Shaoxing, China). *N*-alkane nC6 ∼ nC16 was bought from RESTEK Co., Ltd. (Pennsylvania, USA), Z-Ligustilide (purity ≥ 98%) was purchased from Chengdu Ruifensi Biotechnology Co., Ltd.(Chengdu, China). Ferulic acid (purity ≥ 98%) was purchased from Shanghai Yuanye Biotechnology Co., Ltd. (Chengdu, China). Senkyunolide I (purity ≥ 98%) was purchased from Nanjing Jinyibai Biotechnology Co., Ltd. (Nanjing, China). Phosphoric acid and glacial acetic acid (HPLC-grade) were purchased from Nanjing Chemical Reagent Co., Ltd. (Nanjing, China); Acetonitrile and formic acid (LC/MS-grade) were obtained from Fisher Scientific Co. (Fair Lawn, USA). 1,1-diphenyl-2-picrylhydrazyl (DPPH, purity ≥ 98%) was purchased from Yuanye Bio-Technology Co., Ltd. (Shanghai, China). 2,2′-azino-bis-(3-ethylbenzothiazoline-6-sulfonic acid) (ABTS, purity ≥ 98%) and 2-phenyl-4,4,5,5-tetramethylimidazoline-1-oxyl-3-oxide (PTIO, purity ≥ 98%) were obtained by Aladdin (Shanghai, China). Potassium persulfate (K_2_S_2_O_8_) was purchased from Sinopharm Chemical Reagent Co., Ltd (Shanghai, China). Experimental water was ultra-pure water, and other reagents were analytically pure.

### 2.2 Preparation of RAS, WAS, and WSAS

**Fig. 1:**
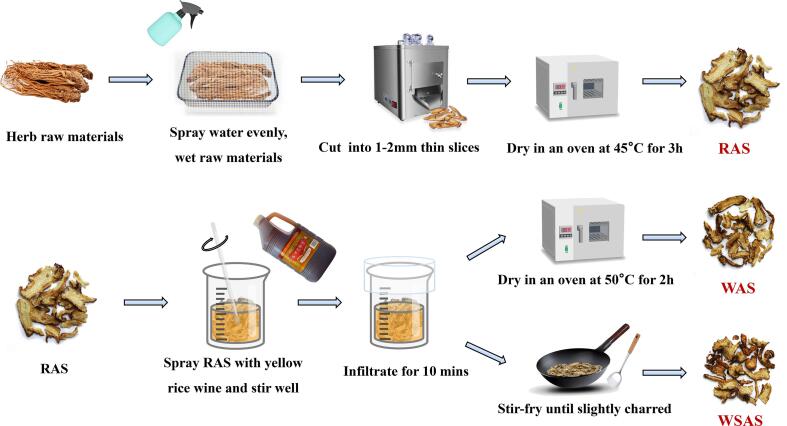
Schematic diagram of the processing for RAS, WAS, and WSAS.

*Angelica sinensis* was purchased from Minxian (Gansu, China) and was authenticated by Prof. Jing Zhou from the Nanjing University of Chinese Medicine. After removing impurities, the AS was moistened with water, then the moistened AS was cut into 1–2 mm slices and dried in an oven at 45 °C for 3 h to obtain RAS. On the basis of the RAS, the RAS was processed with yellow rice wine using different processing methods to obtain WAS and WSAS. A schematic diagram of the processing methods for the RAS, WAS, and WSAS is shown in Fig. 1. Three types of *Angelica sinensis* were powdered with an electrical grinder and passed through a 50-mesh sieve for further analysis.

### 2.3 Detection of color differences in three AS using spectrophotometer

Spectrophotometer CM-5 (Konica Minolta, Japan) was used to detect differences in the color of three AS. After the instrument had been switched on and stabilized with a corrector, the images were obtained by evenly placing the samples on a surface dish and recording each sample's color number and ratio (Esatbeyoglu et al., 2023). Each color number was expressed in terms of L* (lightness), a* (red and green values), b* (yellow and blue values), and the total color value (Feng et al., 2022). Where the total color value of the sample can be expressed as E *_ab_ by the formula E *_ab_=$\sqrt{{\left(L*\right)}^{2}+{\left(a*\right)}^{2}+{\left(b*\right)}^{2}}$, the larger the total color value, the lighter the color. In addition, the full-color difference value ΔE*_ab_ can express the difference between two colors, which is calculated as ΔE *_ab_ = $\sqrt{{\left(\Delta L*\right)}^{2}+{\left(\Delta a*\right)}^{2}+{\left(\Delta b*\right)}^{2}}$.

### 2.4 Detection of aromas differences in three AS using GC E-nose

The three kinds of AS samples were analyzed by Heracles Neo analyzer (Alpha Mos, Toulouse, France), which is a flash GC with a trap, equipped with an embedded odor concentrator. The columns of DB-5 (10 m × 0.18 mm × 0.4 µm) and DB1701 (10 m × 0.18 mm × 0.4 µm) were used together for analysis. The volatile components were separated by both columns and detected with two flame ionization detectors (FID), and the signal was recorded by AlphaSoft Software (Alpha Mos, Toulouse, France).

A single-factor test was carried out on the incubation temperature and time, sample injection volume, and sample dosage to obtain better chromatographic information and analysis results. The final sample analysis parameters were as follows: Samples (0.5 g) were added to a 20 mL headspace bottle and incubated at 70 °C for 20 min with a stirring speed of 500 rpm. The injection volume was 3000 µL with 125 µL/s, and the temperature of the injector was 200 °C. The trapping temperature was 50 °C, the shunt rate of the trap was 10 mL/min, and the capture duration was 26 s. The oven was controlled by the following temperature program: initial temperature 50 °C ramped at 2 °C/s to 120 °C (0 s), then ramped at 0.5 °C/s to 140 °C (0 s), then ramped at 3 °C/s to 250 °C (21 s). The FID detector's temperature was at 200 °C, hydrogen was used as a carrier gas, and the flow rate was 1 mL/min. The same sample was injected six times to verify the repeatability and stability of the instrument and method. The repeatability and stability of instrument and method were measured by the RSD of the characteristic peak area and the relative retention time.

### 2.5 Quantitative determination of ferulic acid

Determination of ferulic acid in three AS by reference to the *Chinese Pharmacopoeia* (Yang et al., 2022b). 200 mg powder of each sample was accurately weighed and refluxed with 20 mL 70% methanol for 0.5 h, cooled, and made up the lost methanol, then centrifuged to obtain the supernatant, filtered with a 0.22 µm membrane, and injected into HPLC for analysis. The ferulic acid standard was dissolved in 70% methanol to a concentration of 12µg/mL as a reference.

HPLC (Waters Co., Ltd. USA) system was used to perform the analysis. A Hedera ODS-25um column (200 mm × 4.6 mm, 5 µm) was operated at 35 °C. The mobile phase and elution parameters were as follow: Acetonitrile and 0.085% phosphoric acid (17:83) eluting for 20 min with a flow rate of 1 mL/min, the injection volume was 10 µL, and the wavelength of detection was 316 nm.

### 2.6 Quantitative determination of Z-ligustilide

Determination of Z-*ligustilide* in three AS by reference to our previous study (Zhang et al., 2020). 200 mg powder of each sample was accurately weighed and ultrasonically extracted with 20 mL 70% methanol for 0.5 h, then centrifuged to obtain the supernatant, filtered with a 0.22 µm membrane, and injected into HPLC for analysis. The Z-ligustilide standard was dissolved in 70% methanol as a reference.

HPLC system and column were identical to the ferulic acid content determination. The mobile phase was composed of acetonitrile (A) −0.2% glacial acetic acid (B) with a flow rate of 1 mL/min at 30 °C. The elution parameters were: 0 ∼ 16 min, 15%∼23% A; 16 ∼ 20 min, 23%∼28% A; 20 ∼ 25 min, 28%∼30% A; 25 ∼ 30 min, 30% A;30 min, 30% A; 30 ∼ 35 min, 30%∼65% A; 35 ∼ 45 min, 65%∼95% A. The injection volume was 20 µL, and the wavelength of detection was 302 nm.

### 2.7 Determination of extracts content

Determination of extracts content in three AS by reference to the *Chinese Pharmacopoeia*. 2.0 g powder of each sample was accurately weighed and immersed in 20 mL 70% ethanol for 1 h. Then the extraction was then refluxed for 1 h, make up the lost ethanol and filtered. The filtrate was accurately measured at 25 mL, dried at 105 °C for 3 h and then cooled in a dryer for 30 min. The alcohol-soluble extract content (%) of the dried test samples was calculated.

### 2.8 UPLC-Q-Orbitrap HRMS/MS analysis

2.0 g powder of each sample was accurately weighed and extracted by ultrasonic with 20 mL 70% methanol for 0.5 h. Made up the lost methanol, then centrifuged to obtain the supernatant, filtered with a 0.22 µm membrane, and injected into UPLC-MS for analysis.

UPLC was performed on an instrument coupled to a Dionex 3000 Ultimate UPLC with an auto-sampler and a Q-Exactive Orbitrap HRMS/MS (Thermo Fisher, MA, USA). An Acquity UPLC BEH C18 (2.1 mm × 100 mm, 1.7 μm) was operated at 30 °C. The mobile phase was: (A) 0.1% formic acid in water, and (B) acetonitrile with a flow rate of 0.3 mL/min. The elution parameters were: 0–1 min, 15% B; 1–15 min, 15%-45% B; 15–25 min, 45%-95% B; 25–26 min, 95% B; 26–27 min, 95%-15% B; 27–30 min, 15% B.

MS analysis was performed with a heated electrospray ionization source in both positive and negative ionization modes, and the mass scan range was *m*/*z* 50–1000. The parameters of positive mode were: auxiliary gas flow, 15 arb; sheath gas flow, 55 arb; spray voltage, 2000 V, auxiliary gas heater temperature, 120 °C; capillary temperature, 450 °C. The parameters of negative mode were: auxiliary gas flow, 15 arb; sheath gas flow, 55 arb; spray voltage, 2500 V, auxiliary gas heater temperature, 120 °C; capillary temperature, 400 °C.

### 2.9 Free radical scavenging assays of three AS *in vitro*

The free radical-scavenging assays were performed to preliminary evaluate and compare the biological activity of three kinds of AS, including DPPH-scavenging assay, ABTS^+^-scavenging assay, and PTIO-scavenging assay. For DPPH-scavenging assay, the DPPH solution (0.04 mg/mL) was mixed with samples (2.5 mg/mL) and incubated at 37 °C in dark for 30 min. The absorbance was measured at λ = 517 nm, and then the scavenging rate was calculated. For ABTS^+^-scavenging assay, ABTS^+^ radicals were prepared by dissolving 8.16 mg of ABTS and 1.4 mg of K_2_S_2_O_8_ in 2 mL distilled water, then 1 mL of each was mixed well and kept away from light for 12 h. Before use, dilute with methanol until its absorbance at 734 nm is 0.700 ± 0.005. Samples (200 µL, 2.5 mg/mL) and ABTS^+^ radical (800 µL) were reacted at 37 °C in the dark for 6 min. The absorbance was detected at 734 nm and the scavenging rate was calculated. For PTIO-scavenging assay, samples (1.5 mL, 10 mg/mL) and PTIO radical (200 µL, 0.188 mg/mL) were incubated at 37 °C in the dark for 2 h. The absorbance was detected at 585 nm and the scavenging rate was calculated.

### 2.10 Statistical analysis

The data was performed by SPSS 22.0 software and expressed as mean ± SEM. One-way ANOVA with LSD test was measured to compare multiple groups, and p-value<0.05 was considered statistically significant.

For UPLC-MS analysis, the raw data was imported into Compound Discoverer 3.1 software (Thermo Fisher Scientific, MA, USA) for peak detection, normalization, and alignment. The possible molecular formula was fitted through the extracted molecular ion chromatographic and isotope peaks. The measured spectrum of secondary fragments was matched with the mz Cloud network database and local TCM composition database. Finally, the filtered ion and compound information in the database was compared with related literature, and the compounds were analyzed and identified.

## 3 Results

### 3.1 The color among the three AS was different in visual sensory and spectrophotometer analysis

**Fig. 2:**
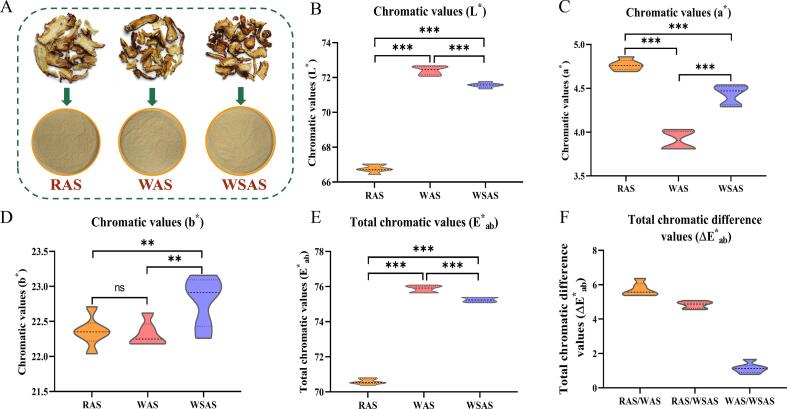
Comparison of chromatic values among RAS, WAS, and WSAS. (A) Powder color comparison under intuitive observation; (B) Chromatic value of L*; (C) Chromatic value of a*; (D) Chromatic value of b*; (E) Total chromatic value of E*_ab_; (F) Total chromatic difference value of ΔE*_ab_ (*p < 0.05; ^**^p < 0.01; ^***^p < 0.001).

The appearance and color of three kinds of AS were compared by naked eye observation and spectrophotometer. Intuitively, the color of RAS slices changed markedly after washing or stir-frying with yellow rice wine processing. WSAS had the most obvious morphological shrinkage, the darkest color, and a slight burning focus. During the stir-frying process, some pieces were broken and with less integrity (Fig. 1). Interestingly, however, after crushing, the powder color of RAS appeared darker than the other two, indicating that RAS not only changed its appearance after processing, but also changed more significantly inside the slices (Fig. 2A). Furthermore, the spectrophotometer detected the color changes of the three AS in more detail and objectively (Boue et al., 2019). The results showed that the L* value and E*_ab_ value of the three kinds of AS were significantly different, and WAS > WSAS > RAS, indicating that the color became lighter after processing in yellow rice wine, and the color of WAS was the lightest (Fig. 2B, E). In addition, the ΔE *_ab_ of the three AS were all greater than 1.0, indicating that the color of them was significantly different (Fig. 2F). In Fig. 2C and D, the a* values of the three AS are all positive and there are significant differences in pairwise comparisons, indicating that their colors are biased towards red, and the color of RAS becomes lighter after yellow rice wine processing. The b* values of the three kinds of AS were also positive and the value of WSAS is significantly higher than RAS and WAS, indicating that the color of RAS was yellower after wine stir-frying, which was consistent with the color change trend of the slices seen by the naked eye.

### 3.2 The aroma among the three AS was different in odor sensory evaluation and chromatograms characterization

**Fig. 3:**
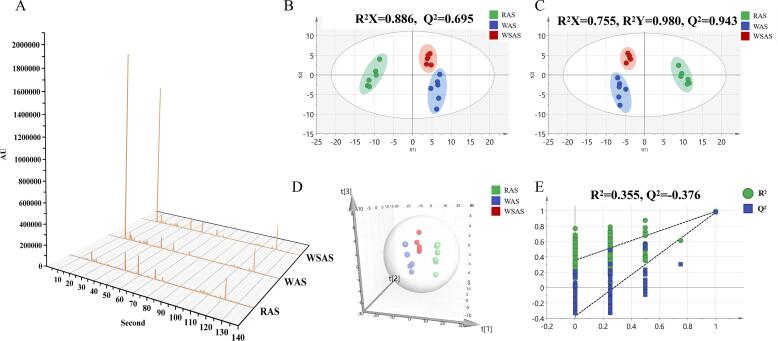
Aroma profiles characterization and multivariate statistical analysis of RAS, WAS, and WSAS. (A) Representative aroma chromatogram; (B) PCA score plots, (C) PLS-DA score plots, (D) PLS-DA (3D) spots, (E) 200X permutation test of PLS-DA mode.

The odor sensory evaluation of the three kinds of AS was carried out. Obviously, the odor of RAS was found to be irritating, softened after the processing of yellow rice wine (WAS and WSAS), and accompanied by the aroma of yellow rice wine. In addition, although the aroma of wine stir-frying was not as strong as that of wine washing, it smelled sweet with a unique burnt aroma. In order to comprehensively and objectively evaluate their different smells, the odor of three kinds of AS was analyzed by Heracles Neo flash GC E-nose, and the representative odor chromatograms of them were shown in Fig. 3A. The relative standard deviations of retention times (0.06–0.11%) and peak areas (1.72–3.48%) of these selected peaks were calculated (Table. S1). Above all, the developed GC E-nose method showed good repeatability and provided good-quality data in this study (Li et al., 2022b). From the chromatogram of the smell, it could be seen that there were obvious differences in the chromatograms of the three kinds of AS, indicating that the aroma components of RAS have changed significantly after processing with yellow rice wine, and the biggest difference in odor peaks was the peak produced by yellow rice wine.

### 3.3 Chemometric statistical analysis of three kinds of AS aroma profiles

SIMCA14.1 was used to analyze the aroma profiles among the RAS, WAS, and WSAS (Ge et al., 2023). The data for the aroma profiles of the RAS, WAS, and WSAS were patterned by an unsupervised PCA method, which indicated that the odor profiles were distinctly different among the RAS, WAS, and WSAS (Fig. 3B). Subsequently, a supervised PLS-DA analysis was performed, and the results suggested that aroma profiles were different among the RAS, WAS, and WSAS, which was in accordance with the PCA results. For the PLS-DA model, the evaluation parameters were R^2^X = 0.755, R^2^Y = 0.980, Q^2^ = 0.943, and 200X permutation testing, with R^2^ = 0.355 and Q^2^ = -0.376, suggesting that PLS-DA model possesses goodness of fit and predictive ability (Fig. 3C, E). Moreover, the 3D PLS-DA spots revealed that the RAS, WAS, and WSAS separated well and clustered distinctly (Fig. 3D). These results indicated that the odor of RAS changed significantly after yellow rice wine processing, which was consistent with the results of chromatogram.

### 3.4 Screen and identification of potential differential aroma components

**Fig. 4:**
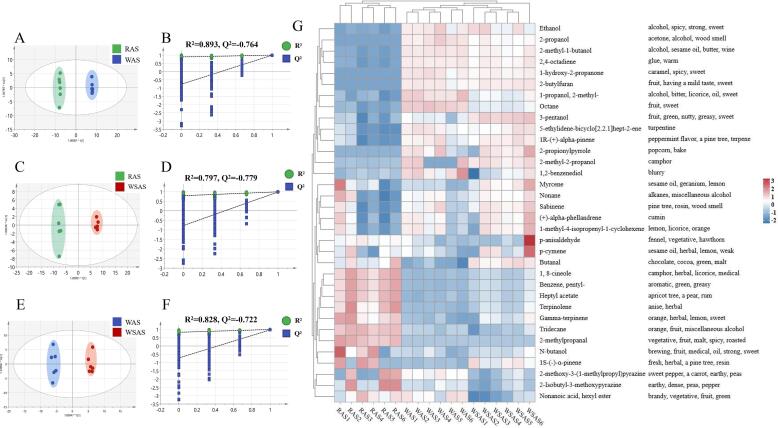
Screening of differential aroma components for RAS, WAS, and WSAS. (A-B) OPLS-DA score plots of RAS and WAS, and 200X permutation test; (C-D) OPLS-DA score plots of RAS and WSAS, and 200X permutation test; (E-F) OPLS-DA score plots of WAS and WSAS, and 200X permutation test; (G) Heat map of odor difference.

OPLS-DA scores were often applied to maximize the groups separation and identify potential differential aroma components (Hu et al., 2021). In Fig. 4A, C, E, the OPLS-DA scores of RAS, WAS, and WSAS pairwise comparisons showed that the aroma profiles were completely separated, indicating there were odor differences between RAS, WAS and WSAS pairwise comparisons. Simultaneously, 200X permutation tests were performed with R^2^ = 0.893 and Q^2^ = -0.764 (RAS vs WAS), R^2^ = 0.797 and Q^2^ = -0.779 (RAS vs WSAS), R^2^ = 0.828 and Q^2^ = -0.722 (WAS vs WSAS), demonstrated a good explanation and prediction for the three OPLS-DA model (Fig. 4B, D, F). Finally, differential aroma compounds were screened based on their VIP value above 1.0 and *p*-values below 0.05. According to the above principle, 34 potential aroma biomarkers in three AS were identified (alcohols, aldehydes, terpenoids, *etc.*) and the heatmap was further used to visualize the different aroma components (Table. S2, Fig. 4G). The results of the heat map showed that most of the aroma components of RAS had the odor of the herb material itself, accompanied by irritation. After the processing of yellow rice wine, WAS and WSAS appeared the smell of wine, and the smell became soft and sweet. Compared with WSAS, WAS retains some of the odors of RAS, while WSAS has more baking-specific odors and oily aromas than WAS. In general, after different processing of RAS in yellow rice wine, the odor of WAS and WSAS became no longer obviously irritating, and new odors were added, which was in line with the characteristics of traditional Chinese medicine processing (Liu et al., 2022).

### 3.5 Determination of ferulic acid, Z-ligustilide and extracts content

**Fig. 5:**
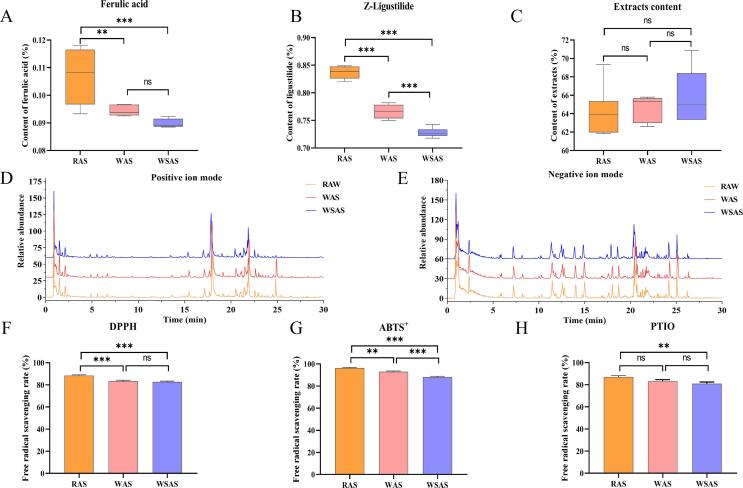
Ferulic acid, Z-ligustilide, and extracts levels determination, chemical profiles characterization, and free radical scavenging ability assessment of RAS, WAS, and WSAS. (A) The level of ferulic acid; (B) The level of Z-ligustilide; (C) The content of extracts; (D) The TIC in positive ion mode; (E) The TIC in negative ion mode; (F) The scavenge rate of DPPH; (G) The scavenge rate of ABTS^+^; (H) The scavenge rate of PTIO (*p < 0.05; ^**^p < 0.01; ^***^p < 0.001).

To explore whether the color and odor difference before and after wine wash and wine stir-flying of RAS were related to the change of internal chemical composition, the content of representative components ferulic acid and Z-ligustilide in AS, and the content of the three AS extracts were determined. Recent researches have showed that ferulic acid and some of the phthalides such as Z-ligustilide and dimeric phthalide are unstable at high temperature (Liang et al., 2014). The results showed that the contents of ferulic acid and Z-ligustilide in WAS and WSAS were significantly lower than those in RAS (Fig. S1 and Fig. 5A, B), and the content of Z-ligustilide in WSAS was also significantly lower than that of WAS. Z-ligustilide is a volatile component with certain irritation. The content of Z-ligustilide in RAS decreased after yellow rice wine processing, suggesting that the decrease of volatile oil components may be closely related to the softening of odor after processing (Fei et al., 2022). Moreover, the difference in Z-ligustilide content between WAS and WSAS may be due to the different temperatures during the two processes, which may also be one of the reasons for their different odors. Notably, there was no significant difference in the extract content of RAS, WAS and WSAS (Fig. 5C), suggesting that RAS after yellow rice wine processing may involve more changes in components and need further analysis.

### 3.6 Global chemical profiling of components in three AS by UPLC-Q-Orbitrap HRMS/MS analysis

To further comprehensively evaluate the differences in the chemical composition of the RAS after processing with wine washing and wine stir-frying, a fast UPLC-Q-Orbitrap HRMS/MS analysis hyphenated technique was first utilized to analyze the chemical profile of the RAS, WAS, and WSAS. The accurate mass and composition of the precursor ions and product ions from the three AS were analyzed in both positive and negative ionization modes. Finally, a total of 85 representative common components (Table 1) and 37 unique components (Table S3) were tentatively identified from the three AS based on comparing retention behavior, HRMS/MS data, and mass fragment characteristics with the compounds in previous references and/or available reference compounds (Chen et al., 2009, Yin et al., 2021). The results showed that *Angelica sinensis* contained a variety of active ingredients, including phenylpropanoids, phthalides, organic acids, fatty acids, and amino acids. The representative total ion chromatography results were displayed in Fig. 5D, E. The peak areas of ion chromatography before and after processing and different processing method groups were integrated, and the data were processed uniformly using the normalization method. In comparison to RAS, the content of phthalide compounds such as Z-ligustilide, E-ligustilide, and 3-butylidenephthalide in RAS and WSAS decreased, while the senkyunolide B and 3-butylphthalide levels increased after yellow rice wine processing. Z-ligustilide is the most abundant active compound in AS and also the main irritant. An excess amount of Z-ligustilide could result in xeransis, nausea, and anesthesia of the oral cavity and tongue, and the appropriate reduction of Z-ligustilide can reduce the irritation (Zhan et al., 2011). Researches have revealed that most effective compounds of AS are unstable and easily decompose or transform. For example, Z-ligustilide readily oxidizes into senkyunolide I and senkyunolide H or polymerizes into levistilide A in air or with heating (Zhang et al., 2019). 3-butylidenephthalide is also unstable, and is easily oxidized and isomerized into 3-butylphthalide (Li et al., 2013). Interestingly, the content of senkyunolide A, senkyunolide I, Levistolide A, and angelicide in WAS was higher than RAS and WSAS, while the content of senkyunolide H, senkyunolide F and confer ferylulate in WSAS was significantly higher than others. In addition, the difference between WAS and WSAS was also reflected in the fact that WAS has more amino acids and phenylpropanoids, while WSAS had more fatty acid components, which were caused by different processing methods of yellow rice wine.

**Table 1:** Common compounds identified from the three AS by UPLC-Q-Orbitrap HRMS/MS.

Mode	No.	Compound	RT	Expected	Measured	Adduct ion	Formula	Delta	MS2 ion	Classification	RAS	WAS	WSAS
			(min)	(*m*/*z*)	(*m*/*z*)			(ppm)	(*m*/*z*)				
**ESI^+^**	1	2-methyl-l-arginine	0.86	188.1273	189.1342	[M + H]±	C7H16N4O2	−2.13	NA	amino acid	1.00	1.04	1.27
	2	Arginine	0.87	174.1117	175.1188	[M + H]±	C6H14N4O2	−1.04	130.0978, 70.0658	amino acid	1.00	1.61	1.09
	3	dl-Histidine	0.88	155.0692	156.0765	[M + H]±	C6H9N3O2	−1.68	NA	amino acid	1.00	1.00	0.97
	4	Glucinol	0.94	342.1162	381.0784	[M + K]^+^	C12H22O11	−1.33	374.5591, 163.3037, 128.1416	others	1.00	0.99	1.01
	5	Tryptophan	1.50	204.0899	205.0968	[M + H]±	C11H12O2N2	−1.87	170.0596, 143.0729, 118.0653, 91.0547	amino acid	1.00	0.99	0.96
	6	Dendryphiellic acid A	2.72	154.0994	155.1065	[M + H]±	C9H14O2	−1.11	NA	fatty acids	1.00	1.12	1.02
	7	Coniferyl aldehyde	3.38	178.0629	179.0700	[M + H]±	C10H10O3	−1.92	118.0414, 107.0494, 91.0547, 65.0393	phenylpropanoids	1.00	0.86	0.89
	8*	Ferulic acid	4.03	194.0579	195.0650	[M + H]±	C10H10O4	−0.69	134.0361, 117.0336, 89.0390, 78.0470, 63.0236	phenylpropanoids	1.00	0.84	0.82
	9	Isoferulic acid	4.58	194.0579	195.0651	[M + H]±	C10H10O4	−0.39	134.0362, 117.0337, 89.0391	phenylpropanoids	1.00	0.90	0.85
	10	Senkyunolide F	5.58	206.0943	207.1021	[M + H]±	C12H14O3	−1.15	169.0649, 152.0618, 128.0620, 79.0547	phthalides	1.00	0.97	1.14
	11*	Senkyunolide I	6.65	224.1049	247.0935	[M + Na]±	C12H16O4	−1.82	170.7626, 105.0701, 91.0545	phthalides	1.00	1.09	0.99
	12	Senkyunolide H	7.20	224.1049	247.0936	[M + Na]±	C12H16O4	−1.90	170.7626, 105.0701, 91.0545	phthalides	1.00	1.06	1.14
	13	7,3′,4′-Trimethoxyflavone	8.19	312.0998	313.1065	[M + H]±	C18H16O5	−1.73	249.1483, 177.0554, 145.0282, 117.0336	flavonoids	1.00	0.96	0.89
	14	3-Butylidenephthalide	9.30	188.0837	189.0907	[M + H]±	C12H12O2	−1.46	145.0645, 128.0620, 79.0548	phthalides	1.00	0.87	0.89
	15	Methoxyeugenol	9.68	194.0943	195.1014	[M + H]±	C11H14O3	−0.82	131.9740, 113.9639, 91.0581, 72.9378	phenylpropanoids	1.00	0.96	1.01
	16	Pseudomonate C	13.66	483.2958	484.3050	[M + H]±	C26H43O8	3.90	294.2059, 235.1325, 207.1376	others	1.00	0.94	0.95
	17	Senkyunolide A	15.48	192.1150	193.1220	[M + H]±	C12H16O2	−1.48	117.0700, 105.0449, 91.0546	phthalides	1.00	1.02	1.01
	18	3-Butylphthalide	16.09	190.0994	191.1063	[M + H]±	C12H14O2	−1.92	155.0597, 115.0544, 91.0546	phthalides	1.00	1.46	1.33
	19	Sedanolide	16.35	194.1306	195.1377	[M + H]±	C12H18O2	−1.16	131.9740, 107.0857, 91.0547, 72.9378	phthalides	1.00	1.00	1.05
	20	E-ligustilide	17.12	190.0994	191.1063	[M + H]±	C12H14O2	−1.92	155.0597, 129.0698, 115.0543, 91.0546	phthalides	1.00	0.98	0.97
	21	Dehydrophytosphingosine	17.70	315.2773	316.2840	[M + H]±	C18H37NO3	−2.15	95.0860, 60.0452	fatty acids	1.00	1.04	0.86
	22*	Z-Ligustilide	17.95	190.0994	191.1062	[M + H]±	C12H14O2	−2.44	129.0701, 117.0703, 115.0544, 103.0544, 91.0546	phthalides	1.00	0.83	0.72
	23	Trimethylolpropane 2-ethylhexanoate laurate	19.13	436.3400	437.3480	[M + H]±	C23H48O7	1.69	437.3479, 175.1188, 158.0923, 81.0705, 70.0658	fatty acids	1.00	1.11	1.14
	24	E-Butylidenephthalide	19.74	188.0837	189.0907	[M + H]±	C12H12O2	−1.46	145.0644, 128.0620, 79.0548	phthalides	1.00	0.87	0.88
	25	Gelispirolide	20.50	380.1988	381.2052	[M + H]±	C24H28O4	−2.27	191.1064, 173.0959	phthalides	1.00	0.86	0.86
	26	Z,Z'-6,8′,7,3′-Diligustilide	20.54	380.1988	381.2052	[M + H]±	C24H28O4	−2.27	191.1064, 173.0959, 145.1011	phthalides	1.00	0.40	0.38
	27	Z-Butylidenephthalide	20.55	188.0837	189.0906	[M + H]±	C12H12O2	−2.10	145.0643, 128.0620, 79.0549	phthalides	1.00	0.79	0.78
	28	1beta-Hydroxycholic acid	20.72	424.2825	425.2889	[M + H]±	C24H40O6	−2.08	345.4098, 121.1012, 95.0860, 81.0704	fatty acids	1.00	1.00	1.09
	29	2-Hydroxylinolenic acid	21.08	294.2195	295.2267	[M + H]±	C18H30O3	−1.53	108.0857, 93.0702, 67.0546	fatty acids	1.00	1.03	1.05
	30	Tokinolide B	21.46	380.1988	381.2052	[M + H]±	C24H28O4	−2.11	191.1064, 173.0959	phthalides	1.00	0.99	0.97
	31	1-Octen-3-yl glucoside	21.64	290.1729	625.3438	[2 M + FA-H]-	C14H24O6	1.32	255.2326, 119.0336, 89.0230	others	1.00	1.00	1.07
	32	Angelicide	21.90	380.1988	381.2052	[M + H]±	C24H28O4	−2.27	191.1065, 173.0961, 145.1012, 117.0701	phthalides	1.00	1.06	1.00
	33	Linoleylglycine	22.57	323.2824	324.2891	[M + H]±	C20H37NO2	−1.81	109.1013, 81.0704, 62.0608	amino acid	1.00	1.06	1.06
	34	Levistolide A	22.71	380.1988	381.2055	[M + H]±	C24H28O4	−1.64	191.1063, 173.0959	phthalides	1.00	1.21	1.05
	35	gaba-linoleamide	22.91	365.2930	366.2996	[M + H]±	C22H39NO3	−1.18	119.0858, 104.0709, 86.0605	amino acid	1.00	0.94	0.95
	36	Z-Ligustilide dimer E232	23.06	380.1988	381.2055	[M + H]±	C24H28O4	−1.38	191.1064, 173.0959, 145.1010	phthalides	1.00	0.48	0.60
	37	Z,Z'-3,3′,8,8′-Diligustilide	23.11	380.1988	381.2055	[M + H]±	C24H28O4	−1.48	191.1064, 173.0958, 91.0546	phthalides	1.00	1.00	0.48
	38	Linoleamide	23.34	279.2562	280.2632	[M + H]±	C18H33NO	−1.11	149.0231, 95.0860, 81.0704, 67.0549	fatty acids	1.00	0.05	0.03
	39	Propyl cinnamate	23.79	190.0994	191.1065	[M + H]±	C12H14O2	−1.03	129.0700, 115.0543, 91.0547	fatty acids	1.00	1.41	1.56
	40	Palmitic amide	24.45	255.2562	256.2629	[M + H]±	C16H33NO	−1.15	173.4406, 102.0916, 88.0761	phenylpropanoids	1.00	0.83	0.32
	41	Oleamide	24.81	281.2719	282.2786	[M + H]±	C18H35NO	−2.06	121.1014, 93.0702, 83.0860, 69.0705	fatty acids	1.00	1.13	0.16
	42	Plastoquinone 3	25.40	340.2402	341.2469	[M + H]±	C23H32O2	−1.81	191.1064, 173.0958, 91.546	terpenoids	1.00	0.72	0.53
	43	Stearamide	26.38	283.2875	284.2943	[M + H]±	C18H37ON	−1.59	116.1069, 102.0916, 88.0761	fatty acids	1.00	0.40	0.19
**ESI^-^**	1	Gluconic acid	0.96	196.0583	195.0502	[M−H]^-^	C6H12O7	1.24	160.9968, 99.9060, 75.0073	organic acids	1.00	1.04	0.99
	2	Malic acid	1.03	134.0215	133.0129	[M−H]^-^	C4H6O5	−1.73	124.7240	organic acids	1.00	1.09	1.04
	3	Citric Acid	1.23	192.0270	191.0188	[M−H]-	C6H8O7	0.74	NA	organic acids	1.00	0.87	0.93
	4	Chlorogenic acid	1.57	354.0951	353.0875	[M−H]-	C16H18O9	−1.07	325.0900, 191.0553, 135.0436, 85.0281	organic acids	1.00	0.83	0.91
	5	Histamine-betaxanthin	1.80	304.1172	305.1238	[M−H]-	C14H16N4O4	−1.81	305.1225, 200.0703, 148.0391, 71.0498	amino acid	1.00	1.09	1.20
	6	Caffeic acid	2.19	180.0423	179.0341	[M−H]-	C9H8O4	0.87	134.0361	phenylpropanoids	1.00	0.93	1.14
	7	Vanillic acid	2.38	168.0423	167.0339	[M−H]-	C8H8O4	−0.09	183.5801, 152.1316	phenylpropanoids	1.00	0.96	0.90
	8	Leucinic acid	2.92	132.0786	131.0701	[M−H]-	C6H12O3	−1.30	NA	amino acid	1.00	0.96	1.07
	9	4-Oxododecanedioic acid	4.84	244.1311	243.1235	[M−H]-	C12H20O5	−1.13	NA	organic acids	1.00	0.94	0.98
	10	Isochlorogenic acid C	5.82	516.1268	515.1195	[M−H]-	C25H24O12	−0.17	353.0876, 191.0553, 173.0445	organic acids	1.00	0.89	1.05
	11	Azelaic acid	5.93	188.1049	187.0968	[M−H]-	C9H16O4	1.52	125.0957, 97.0642, 61.9868	organic acids	1.00	1.01	1.14
	12	Acrimarine H	6.15	513.1788	512.1705	[M−H]-	C30H27NO7	2.00	306.0766, 160.0064, 143.0452, 127.0501, 74.0233	alkaloids	1.00	0.82	0.89
	13	3,4,5-Trimethoxycinnamic acid	7.23	238.0841	237.0762	[M−H]-	C12H14O5	2.11	193.0862, 109.0282	organic acids	1.00	1.05	1.13
	14	Acetylshikonin	8.23	330.1103	329.1030	[M−H]-	C18H18O6	3.06	193.0501, 160.0155, 135.0440, 93.0332	quinone	1.00	0.92	0.87
	15	Eugenitin	9.99	220.0736	219.0657	[M−H]-	C12H12O4	2.30	201.4769, 147.0803, 69.0332	phenylpropanoids	1.00	0.92	0.88
	16	Coniferyl ferulate	10.24	356.1260	355.1186	[M−H]-	C20H20O6	2.66	296.1046, 281.0817, 159.0441, 135.0440, 93.0331	phenylpropanoids	1.00	1.04	1.10
	17	Pinellic acid	11.53	330.2406	329.2331	[M−H]-	C18H34O5	2.46	211.1332, 171.1017, 139.1116, 99.0802	fatty acids	1.00	0.74	1.24
	18	Isoeugenyl acetate	11.73	206.0943	205.0863	[M−H]-	C12H14O3	1.70	205.0862, 161.0959, 135.0076, 95.0124	phenylpropanoids	1.00	0.26	0.27
	19	Isoeugenitin	12.56	220.0736	219.0657	[M−H]-	C12H12O4	2.35	219.0662, 189.0186, 176.0105, 132.0204, 93.0695	phenylpropanoids	1.00	0.94	1.02
	20	Z-6,7-Epoxyligustilide	12.78	206.0943	205.0863	[M−H]-	C12H14O3	1.70	161.0963, 131.0489, 106.0410	phthalides	1.00	0.98	1.00
	21	Senkyunolide E	14.05	204.0786	203.0706	[M−H]-	C12H12O3	1.62	173.0233, 160.0156, 132.0205, 95.0126	phthalides	1.00	1.00	1.01
	22	9-Octadecenedioic acid	14.48	312.2301	311.2229	[M−H]-	C18H32O4	3.90	311.2214, 293.2127, 183.1379, 171.1018, 113.0959	fatty acids	1.00	0.98	0.63
	23	O-Ferulic acid	14.72	194.0579	193.0498	[M−H]-	C10H10O4	1.58	133.0283, 89.0382	phenylpropanoids	1.00	0.92	1.00
	24	Senkyunolide B	14.99	204.0786	203.0705	[M−H]-	C12H12O3	1.28	174.0313, 130.0411, 91.0175	phthalides	1.00	1.03	1.03
	25	Licoricone	16.84	382.1416	381.1341	[M−H]-	C22H22O6	2.09	337.0716, 323.0564, 257.0815, 232.0734, 135.0438	Flavonoids	1.00	1.01	1.14
	26	Octadecanedioic acid	17.60	314.2457	313.2382	[M−H]-	C18H34O4	3.11	295.0281, 252.0312	organic acids	1.00	0.97	1.05
	27	12,13-dihydroxyoleic acid	18.05	314.2457	313.2384	[M−H]-	C18H34O4	3.50	300.7622, 277.2177	fatty acids	1.00	1.04	1.24
	28	9Z-Octadecenedioic acid	18.40	312.2301	311.2227	[M−H]-	C18H32O4	3.32	265.2175, 211.1333, 171.1010, 113.0957	fatty acids	1.00	0.97	1.04
	29	Ferulsinaic acid	18.70	398.2093	397.2017	[M−H]-	C24H30O5	1.79	205.0863, 163.1118	organic acids	1.00	0.99	1.03
	30	17-Hydroxylinolenic acid	19.29	294.2195	293.2122	[M−H]-	C18H30O3	2.85	205.6451, 71.0120	fatty acids	1.00	0.58	1.78
	31	Vismione D	19.62	410.2093	409.2018	[M−H]-	C25H30O5	2.10	238.0636, 217.1016, 203.0864, 111.0074	aromatic compounds	1.00	1.02	0.89
	32	9-Hydroxylinoleic acid	19.85	296.2351	295.2277	[M−H]-	C18H32O3	1.01	290.8770, 277.2173, 209.1541	fatty acids	1.00	1.01	1.15
	33	Coriolic acid	20.49	296.2351	295.2275	[M−H]-	C18H32O3	2.50	295.2291, 277.2172, 195.1378, 113.0956	fatty acids	1.00	1.01	1.08
	34	(10E,12Z)-9-Oxooctadeca-10,12-dienoic acid	21.51	294.2195	293.2122	[M−H]-	C18H30O3	3.85	293.2137, 185.1171, 125.0959	fatty acids	1.00	1.01	1.05
	35	20beta-Hydroxyscutione	21.75	434.2457	433.2360	[M−H]-	C28H34O4	−3.11	152.9947, 78.9576	triterpenoids	1.00	0.94	0.59
	36	alpha-dimorphecolic acid	23.10	296.2351	295.2277	[M−H]-	C18H32O3	3.21	249.2220, 141.1270, 83.0488	organic acids	1.00	1.03	1.03
	37	Ganodernoid C	23.65	468.2512	467.2437	[M−H]-	C28H36O6	1.98	282.0898, 241.0864, 212.0474, 71.0487	triterpenoids	1.00	1.12	1.12
	38	*N*-myristoyl leucine	23.96	341.2930	340.2854	[M−H]-	C20H39NO3	2.38	296.2957, 254.2488, 102.0546	amino acid	1.00	1.06	0.98
	39	3-Hydroxyhexadecanoic acid	24.19	272.2351	271.2277	[M−H]-	C16H32O3	3.39	225.2217, 197.1902	fatty acids	1.00	0.95	0.98
	40	2,2′-Methylenebis(4-methyl-6-*tert*-butylphenol)	24.48	340.2402	339.2327	[M−H]-	C23H32O2	2.49	163.1117, 147.1803, 60.5246	aromatic compounds	1.00	1.04	0.35
	41	Linoleic acid	25.07	280.2402	279.2327	[M−H]-	C18H32O2	2.91	279.2331, 239.6518, 121.1610	fatty acids	1.00	1.09	0.85
	42	Palmitic acid	26.20	256.2402	255.2326	[M−H]-	C16H32O2	3.07	232.1399, 136.1028	fatty acids	1.00	0.82	0.80

The compounds marked with “*” were identified by the reference compounds.

### 3.7 Free radical scavenging ability of three AS *in vitro*

The comparison and biological activity of RAS, WAS, and WSAS were further explored using various free radical-scavenging assays, including DPPH-scavenging, ABTS^+^-scavenging, and PTIO-scavenging. These free radical-scavenging assays are commonly used to evaluate the antioxidant capacity of natural products or extracts, and the scavenging reactions may be regarded as a direct antioxidant process (Marinaccio et al., 2023, Wang et al., 2022). DPPH and ABTS^+^ are nitrogen-centered radicals, while PTIO is an oxygen-centered radical, and their differences lead to different scavenging mechanisms (Li et al., 2017). As shown in Fig. 5F-G, the DPPH and ABST^+^ free radical scavenging rates of RAS were significantly higher than those of WAS and WSAS. WAS and WSAS had the same ability to scavenge DPPH free radicals, but WAS was significantly better than WSAS at scavenging ABST^+^ free radicals. Moreover, in PTIO free radical-scavenging assays, the free radical scavenging rate of WAS and WSAS is lower than that of RAS, and there was a significant difference between RAS and WSAS. Although the free radical scavenging rate of WAS is higher than that of WSAS, it is insignificant (Fig. 5H). The differences in free radical scavenging ability among RAS, WAS, and WSAS may explain the mechanism of *Angelica sinensis* processing with yellow rice wine to alleviate drug properties and the reasons for the application in different diseases before and after yellow rice wine processing. It also proved that WAS may have higher antioxidant activity than WSAS *in vitro*, suggesting *Angelica sinensis* prepared by different processing methods of yellow rice wine (WAS and WSAS) could not be confused.

## 4 Conclusion

In the current study, we compared RAS, WAS and WSAS for the first time in terms of color, aroma, chemical composition, and free radical scavenging ability. We found that the RAS undergoes significant changes in color, aroma, components, and the ability of scavenging free radical after processing with yellow rice wine, and the different processing methods also result in significant differences between WAS and WSAS. Nevertheless, future research will call for more experimental data to further explore the differences between WAS and WSAS, such as cell experiments *in vitro* and/or animal studies *in vivo* to compare their efficacy. This study will provide a scientific basis for the rational clinical use of *Angelica Sinensis* and provide a reference for research on the principles of *Angelica Sinensis* processing with yellow rice wine.

## CRediT authorship contribution statement

**Zhi-Tong Zhang:** Methodology, Investigation, Data curation, Writing – original draft. **Yue Jiang:** Methodology, Investigation, Data curation. **Yali Qi:** Methodology, Investigation. **Huanhuan Guan:** Resources. **Lei Bai:** Resources. **Pan Chen:** Investigation. **Wufeng Gao:** Resources. **Guo-Dong Zhuang:** Methodology. **Tulin Lu:** Supervision, Funding acquisition. **Guojun Yan:** Conceptualization, Funding acquisition, Writing – review & editing.

## Declaration of Competing Interest

The authors declare that they have no known competing financial interests or personal relationships that could have appeared to influence the work reported in this paper.

## Data Availability

Data will be made available on request.
