# Supplementary material for: Comparative study on Angelica sinensis after different processing with yellow rice wine in color, aromas, chemical components, and antioxidant activities
Source: Food Chem X. 2023 Aug 6;19:100822. doi: 10.1016/j.fochx.2023.100822 (PMC10534152; doi:10.1016/j.fochx.2023.100822)
Supplement: Supplementary data 1 [file mmc1.doc]

**Supporting Information**

**Comparative Study on *Angelica Sinensis* after Different Processing with Yellow Rice Wine in Color, Aromas, Chemical Components, and Antioxidant Activities**

Zhi-Tong Zhang^1#^, Yue Jiang^1#^, Yali Qi^1^, Huanhuan Guan^1^, Lei Bai^1^, Pan Chen^1^, Wufeng Gao^1^, Guo-Dong Zhuang^2^, Tulin Lu^1^, Guojun Yan^1*^

***Affiliations***

*^1^School of Pharmacy, Nanjing University of Chinese Medicine, Jiangsu Engineering Research Center for Development and Application of External Drugs in Traditional Chinese Medicine, Jiangsu Province Engineering Research Center of Classical Prescription, Nanjing 210023, China*

*^2^Key Laboratory of Digital Quality Evaluation of Chinese Materia Medica of State Administration of TCM and Engineering & Technology Research Center for Chinese Materia Medica Quality of Guangdong Province, Guangdong Pharmaceutical University, Guangzhou 510006, China*

**^*^*Corresponding authors***

Guojun Yan, E-mail: yanguojun@njucm.edu.cn

**^#^** Authors contributed equally to this work.

**Table S1** The RSD of retention times and peak areas

| **Peak. no** | **RT (sec)** | **Retention times**  **RSD (%)** | **Peak areas**  **RSD (%)** |
| --- | --- | --- | --- |
| 1 | 19.81 | 0.11 | 3.48 |
| 2 | 23.68 | 0.11 | 2.55 |
| 3 | 47.71 | 0.07 | 3.02 |
| 4 | 58.27 | 0.06 | 1.72 |
| 5 | 66.28 | 0.06 | 2.51 |
| 6 | 71.34 | 0.06 | 2.39 |
| 7 | 77.03 | 0.06 | 1.89 |


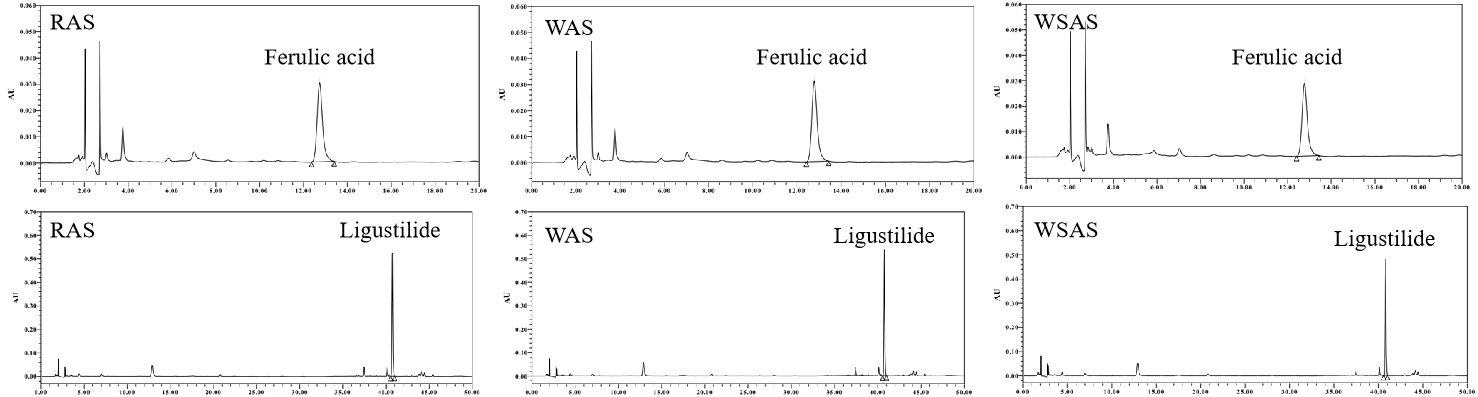


**Fig S1** HPLC chromatogram of content determination

**Table S2** Potentially different aroma components of three AS identified by GC E-nose

| **No.** | **Formula** | **Compound** | **Classification** | **Odor description** |
| --- | --- | --- | --- | --- |
| 1 | C2H6O | Ethanol | alcohols | alcohol, spicy, strong, sweet |
| 2 | C3H8O | 2-propanol | alcohols | acetone, alcohol, wood smell |
| 3 | C4H10O | 2-methyl-2-propanol | alcohols | camphor |
| 4 | C4H8O | 2-methylpropanal | aldehydes | vegetative, fruit, malt, spicy, roasted |
| 5 | C4H8O | Butanal | aldehydes | chocolate, cocoa, green, malt |
| 6 | C4H10O | 1-propanol, 2-methyl- | alcohols | alcohol, bitter, licorice, oil, sweet |
| 7 | C4H10O | N-butanol | alcohols | brewing, fruit, medical, oil, strong, sweet |
| 8 | C3H6O_2_ | 1-hydroxy-2-propanone | ketones | caramel, spicy, sweet |
| 9 | C5H12O | 3-pentanol | alcohols | fruit, green, nutty, greasy, sweet |
| 10 | C5H12O | 2-methyl-1-butanol | alcohols | alcohol, sesame oil, butter, wine |
| 11 | C8H18 | Octane | alkanes | fruit, sweet |
| 12 | C8H14 | 2,4-octadiene | olefin | glue, warm |
| 13 | C8H12O | 2-butylfuran | heterocyclic compounds | fruit, having a mild taste, sweet |
| 14 | C9H20 | Nonane | alkanes | alkanes, miscellaneous alcohol |
| 15 | C9H12 | 5-ethylidene-bicyclo[2.2.1]hept-2-ene | bridged-ring compounds | turpentine |
| 16 | C10H16 | 1R-(+)-alpha-pinene | terpenes | peppermint flavor, a pine tree, terpene |
| 17 | C10H16 | 1S-(-)-ɑ-pinene | terpenes | fresh, herbal, a pine tree, resin |
| 18 | C10H16 | Sabinene | terpenes | pine tree, rosin, wood smell |
| 19 | C10H16 | Myrcene | terpenes | sesame oil, geranium, lemon |
| 20 | C10H16 | (+)-alpha-phellandrene | olefin | cumin |
| 21 | C10H16 | 1-methyl-4-isopropenyl-1-cyclohexene | terpenes | lemon, licorice, orange |
| 22 | C10H14 | p-cymene | terpenes | sesame oil, herbal, lemon, weak |
| 23 | C10H18O | 1, 8-cineole | terpenes | camphor, herbal, licorice, medical |
| 24 | C10H16 | Gamma-terpinene | terpenes | orange, herbal, lemon, sweet |
| 25 | C7H9NO | 2-propionylpyrrole | ketones | popcorn, bake |
| 26 | C10H16 | Terpinolene | terpenes | anise, herbal |
| 27 | C9H18O2 | Heptyl acetate | carboxylic ester | apricot tree, a pear, rum |
| 28 | C11H16 | Benzene, pentyl- | aromatic compound | aromatic, green, greasy |
| 29 | C9H14N2O | 2-methoxy-3-(1-methylpropyl)pyrazine | heterocyclic compounds | sweet pepper, a carrot, earthy, peas |
| 30 | C9H14N2O | 2-Isobutyl-3-methoxypyrazine | heterocyclic compounds | earthy, dense, peas, pepper |
| 31 | C8H8O2 | p-anisaldehyde | aldehydes | fennel, vegetative, hawthorn |
| 32 | C13H28 | Tridecane | alkanes | orange, fruit, miscellaneous alcohol |
| 33 | C6H6O2 | 1,2-benzenediol | phenols | blurry |
| 34 | C15H30O2 | Nonanoic acid, hexyl ester | fatty acid ester | brandy, vegetative, fruit, green |

**Table S3** Unique compounds identified from the three AS by UPLC-Q-Orbitrap HRMS/MS

| **Mode** | **No.** | **Compound** | **RT** | **Expected** | **Measured** | **Adduct ion** | **Formula** | **Delta** | **MS^2^ ion** | **Classification** | **RAS** | **WAS** | **WSAS** |
| --- | --- | --- | --- | --- | --- | --- | --- | --- | --- | --- | --- | --- | --- |
|  |  |  | **(min)** | **(m/z)** | **(m/z)** |  |  | **(ppm)** | **(m/z)** |  |  |  |  |
| **ESI^+^** | 1 | L-Pyroglutamic acid | 0.30 | 129.0426 | 130.0499 | [M+H]^+^ | C_5_H_7_NO_3_ | 0.09 | 84.0814, 70.0659, 56.0503 | amino acid | - | - | + |
|  | 2 | 3-Butylidene-4,5-dihydrophthalide | 0.35 | 190.0994 | 191.1063 | [M+H]^+^ | C_12_H_14_O_2_ | -0.35 | 115.0543, 103.0545, 91.0546, 79.0548 | phthalides | + | + | - |
|  | 3 | Valine | 0.43 | 117.0790 | 150.1124 | [M+H+CH_3_OH]^+^ | C_5_H_11_NO_2_ | -0.64 | 117.9942, 72.1220, 55.2340 | amino acid | - | + | + |
|  | 4 | 3-amino-3-deoxy-D-glucose | 0.89 | 179.0793 | 180.0859 | [M+H]^+^ | C_6_H_13_NO_5_ | -1.38 | NA | sugars | - | + | - |
|  | 5 | Conagenin | 1.01 | 249.1212 | 250.1282 | [M+H]^+^ | C_10_H_19_NO_6_ | -1.32 | NA | amino acid | - | + | - |
|  | 6 | Valylisoleucine | 1.51 | 230.1630 | 231.1699 | [M+H]^+^ | C_11_H_22_N_2_O_3_ | -1.79 | 89.0969, 72.814, 55.0550 | amino acid | - | + | + |
|  | 7 | Monascumic acid | 1.59 | 215.1157 | 198.1125 | [M+H-H_2_O]^+^ | C_10_H_17_NO_4_ | -0.21 | NA | amino acid | - | + | - |
|  | 8 | N-Acetyl-L-glutamate 5-semialdehyde | 1.91 | 173.0688 | 174.0759 | [M+H]^+^ | C_7_H_11_NO_4_ | -0.92 | 86.0606, 69.0340, 43.0184 | amino acid | + | + | - |
|  | 9 | Flavipucine | 2.86 | 237.1001 | 238.1072 | [M+H]^+^ | C_12_H_15_NO_4_ | -0.68 | NA | others | - | + | - |
|  | 10 | 2-Phenylethyl acetate | 3.46 | 164.0837 | 165.0909 | [M+H]^+^ | C_10_H_12_O_2_ | -1.35 | 103.0545, 91.0495, 79.0548 | phthalides | + | + | - |
|  | 11 | Phenylpropiolic acid | 5.83 | 146.0368 | 147.0439 | [M+H]^+^ | C_9_H_6_O_2_ | -0.94 | 147.0435, 129.0330, 68.9965 | phenylpropanoids | + | + | - |
|  | 12 | Setarin | 7.04 | 202.0630 | 203.0700 | [M+H]^+^ | C_12_H_10_O_3_ | -1.3 | 128.0621, 91.0547, 72.9378, 55.9352 | phthalides | - | + | - |
|  | 13 | Cyclo-(Leu-Ile) | 7.08 | 226.1681 | 227.1752 | [M+H]^+^ | C_12_H_22_N_2_O_2_ | -0.83 | NA | others | - | + | + |
|  | 14 | Paneolic acid | 8.99 | 264.1362 | 247.1328 | [M+H-H_2_O]^+^ | C_15_H_20_O_4_ | -1.72 | 180.0351, 161.0957, 123.0799 | Terpenoids | - | - | + |
|  | 15 | Tocopheronic acid | 10.01 | 294.1467 | 295.1537 | [M+H]^+^ | C_16_H_22_O_5_ | -1.153 | 249.1483, 207.1014, 95.0495, 71.0498 | Terpenoids | + | + | - |
|  | 16 | Vinyl caffeate | 10.29 | 206.0579 | 207.0649 | [M+H]^+^ | C_11_H_10_O_4_ | -1.22 | 119.0493, 103.0545, 91.0546, 84.9602 | organic acids | + | + | - |
|  | 17 | Allyl cinnamate | 11.80 | 188.0837 | 189.0907 | [M+H]^+^ | C_12_H_12_O_2_ | -1.32 | 133.0284, 128.0620, 105.0337, 91.0546 | phenylpropanoids | - | + | - |
|  | 18 | Dihydrojasmonic acid | 12.77 | 212.1412 | 213.1482 | [M+H]^+^ | C_12_H_20_O_3_ | -1.47 | 167.0605, 151.0151 | Fatty acids | + | + | - |
|  | 19 | Methyl phenylacetate | 15.47 | 150.0681 | 151.07513 | [M+H]^+^ | C_9_H_10_O_2_ | -0.94 | 116.9722, 105.0338, 95.0494, 57.9356 | phenylpropanoids | + | - | - |
|  | 20 | 4-methyl-6-phenyl-5,6-dihydro-2H-pyran-2-one | 17.53 | 188.0837 | 189.09068 | [M+H]^+^ | C_12_H_12_O_2_ | -1.71 | 128.0620, 115.0544, 91.0546 | others | + | - | - |
|  | 21 | Isopaxidal | 19.15 | 378.1831 | 379.1900 | [M+H]^+^ | C_24_H_26_O_4_ | -1.01 | 189.0906, 161.0958, 143.0853 | Terpenoids | + | - | - |
|  | 22 | Di-4-coumaroylputrescine | 19.40 | 380.1747 | 381.1820 | [M+H]^+^ | C_22_H_24_N_2_O_4_ | 2.95 | 194.1064, 173.0957, 91.0546 | organic acids | - | - | + |
|  | 23 | 1-Phenylheptane-1,5-dione | 23.83 | 204.1150 | 205.1223 | [M+H]^+^ | C_13_H_16_O_2_ | -1.54 | 205.1226, 189.0921 | phenylpropanoids | - | - | + |
|  | 24 | 3-deoxyaphidicolin | 24.40 | 322.2508 | 345.2395 | [M+Na]^+^ | C_20_H_34_O_3_ | -1.53 | NA | Terpenoids | - | + | - |
|  | 25 | (2E,4E,14E)-13-Hydroperoxy-N-(2-methylpropyl)icosa-2,4,14-trienamide | 24.98 | 393.3242 | 394.3308 | [M+H]^+^ | C_24_H_43_NO_3_ | -1.21 | 132.1018, 86.0969 | Alkaloids | - | - | + |
|  | 26 | Pipericine | 25.73 | 335.3188 | 336.3252 | [M+H]^+^ | C_22_H_41_NO | -2.22 | 142.1226, 81.0705, 74.0971, 57.0707 | Alkaloids | - | - | + |
| **ESI^-^** | 1 | Lactulose | 2.61 | 342.1162 | 387.11453 | [M+FA-H]^-^ | C_12_H_22_O_11_ | 0.36 | 179.0547, 119.0337, 89.0230 | sugars | + | - | - |
|  | 2 | Sucrose | 4.46 | 342.1162 | 387.1146 | [M+FA-H]^-^ | C_12_H_22_O_11_ | 3.133 | 341.1085, 179.0554, 119.0336, 89.0230 | sugars | + | - | - |
|  | 3 | Peniisocoumarin G | 7.28 | 252.0634 | 251.0557 | [M-H]^-^ | C_12_H_12_O_6_ | -1.53 | 179.0704, 109.0282 | phthalides | - | - | + |
|  | 4 | Citreodiol | 9.38 | 214.1205 | 213.1127 | [M-H]^-^ | C_11_H_18_O_4_ | -2.38 | 177.9023, 92.9944, 61.9869 | others | - | + | + |
|  | 5 | Phomolactone B | 9.89 | 222.0892 | 221.0815 | [M-H]^-^ | C_12_H_14_O_4_ | -2.04 | 177.0911, 134.0361 | phthalides | - | + | + |
|  | 6 | Ethyl salvianolate | 10.66 | 522.1526 | 521.14569 | [M-H]^-^ | C_28_H_26_O_10_ | 2.81 | 282.0896, 135.0439 | others | - | + | + |
|  | 7 | Auranetin | 11.48 | 372.1209 | 371.11337 | [M-H]^-^ | C_20_H_20_O_7_ | -0.93 | 205.0863, 121.0282 | Flavonoids | + | - | + |
|  | 8 | N-Lactoylisoleucine | 12.93 | 202.1205 | 201.1127 | [M-H]^-^ | C_10_H_18_O_4_ | -1.71 | 199.1691, 139.1113, 92.9244 | organic acids | - | - | + |
|  | 9 | Mycestericin E | 18.14 | 385.2828 | 384.2757 | [M-H]^-^ | C_21_H_39_NO_5_ | 0.49 | NA | Fatty acids | - | - | + |
|  | 10 | Mangiferic acid | 22.32 | 280.2402 | 279.2329 | [M-H]^-^ | C_18_ H_32_O_2_ | -0.26 | 279.2337, 247.3587, 95.0940 | Fatty acids | - | - | + |
|  | 11 | 4,5-ditridecyl-octanedioic acid | 26.26 | 538.4961 | 537.4889 | [M-H]^-^ | C_34_H_66_O_4_ | -0.11 | NA | organic acids | - | + | + |
